# Dairy cow and calf behavior and productivity when maintained together on a pasture-based system

**DOI:** 10.5713/ab.22.0135

**Published:** 2022-06-30

**Authors:** Sarah E. Mac, Sabrina Lomax, Cameron E. F. Clark

**Affiliations:** 1 Livestock Production and Welfare Group, School of Life and Environmental Sciences, Faculty of Science, The University of Sydney, Camden, NSW 2570, Australia

**Keywords:** Calf Development, Cattle-maternal-filial Bond, Maternal Separation, Meat Quality, Vocalization

## Abstract

**Objective:**

We determined the impact of maintaining pasture-based dairy cows and calves together over 100 days on cow milk production, cow and calf behavior, and calf liveweight and carcass quality.

**Methods:**

Six Holstein-Friesian cows and their male calves were monitored for 106±8.6 days. Cows were temporarily separated twice a day for milking with calves remaining in the paddock. Cow and calf behaviors were recorded via scan sampling at 6 different timepoints, for the first 7 days and twice a week thereafter. Calves were weighed weekly and immediately processed for meat quality and rumen development analysis at 106±8.6 days. Daily cow milk yields were collected from enrollment until 109±8.6 days (3 days post-weaning).

**Results:**

The average daily gain of calves was 1.4±0.73 kg/d, with an average carcass dressing percentage of 59%. Calves had the greatest frequency of observed close proximity to cow and suckling in the first two weeks and decreased with experiment duration. During separation for milking, cow vocalizations and attempts to return to their calf decreased over time. Reticulorumen weight was on target for calf age, but as a proportion of total stomach weight was lower than industry averages of calves the same age due to the larger abomasum. Cows produced an average of 12±7.6 kg of milk yield per day over the 3-days before the calves were weaned and increased to mean of 31±8.3 kg/d the 3 days after weaning, indicating a consumption of close to 20 kg per calf per day.

**Conclusion:**

The impact of a pasture-based cow-calf rearing system on cow and calf behavior and the potential for high levels of calf liveweight gain when provided *ad-libitum* milk and feed were determined. Further research is required to determine the practicality of replicating such systems with large herds and impact on reared calves post-weaning.

## INTRODUCTION

Dairy calves are typically removed from their mother soon after birth and artificially reared either individually or in group housing. The practice of early cow-calf separation has become a source of public concern associated with the removal natural behaviors of nursing and suckling [1]. Research examining cow-calf rearing has focused on indoor housing systems [2] with a paucity of data available for pasture-based cow-calf systems with no data available on milk production, calf development, and behavior. Pasture-based systems present new challenges including cows required to walk long distances to the milking parlor and rotational grazing of paddocks which involve daily, sometimes hourly shifts. There are additional concerns regarding calf exposure to the weather, practicality of labor, and practicality of implementation of pasture-based cow-calf systems [3].

Many dairies offer calves a restricted diet of 4 to 6 L milk/d [4] allocated in 2 feeds, but some farms have adopted ‘accelerated’ feeding programs offering greater than 8 L milk/d [5]. Greater quantities of milk increase weight gain [6,7] however, greater milk consumption can decrease solid feed consumption [7] and has been argued to be linked to reduced rumen development [8]. Cow-calf contact systems allow calves access to relatively high volumes of milk, and calves kept with the cow typically show greater rates of body weight (BW) gain, higher milk yields in first lactation [9], but the effects on cow milk production are unclear [10].

Although previous work has assessed the impact of per manent cow-calf separation on cow and calf distress responses [10], cow-calf rearing systems also require periods of temporary separation, for example when cows go to the milking parlor. Research on temporary separation is limited and has focused on motivation and reuniting cows and calves instead of the process of separation. Thus, there is need for work investigating the impact of short-term separation given cows are milked on average twice daily and the potential for a smooth transition to permanent seperation. The objectives of our work were to determine the impact of a pasture-based cow-calf rearing system across an extended duration of 100 days on i) cow milk production, ii) calf BW gains and carcass quality, and iii) cow and calf behavior during separation for milking.

## MATERIALS AND METHODS

### Animal management and monitoring

This experiment was conducted at The University of Sydney’s commercial dairy farm “Corstorphine” between March and August 2019 in accordance with the University of Sydney Animal Ethics Committee regulations (Protocol 2018/1462).

Six multiparous Holstein-Friesian cows (Lactation 3.5±1.4) that calved within a 16 d period were enrolled in the study. Enrolment criteria required cows to have given birth to a male calf with the ability to suckle unassisted after 3 days, and experimenters had to be able to handle calves without aggression from the cow. One cow-calf pair was replaced with another after 4 d due to the calf not suckling unassisted. Due to the nature of the study, male calves were selected over females with the intention of processing the calves post-weaning and harvesting the rumens. Additionally, there was uncertainty of deleterious effects on future health or production of replacement heifers if female calves were used in the study.

**Figure 1:**
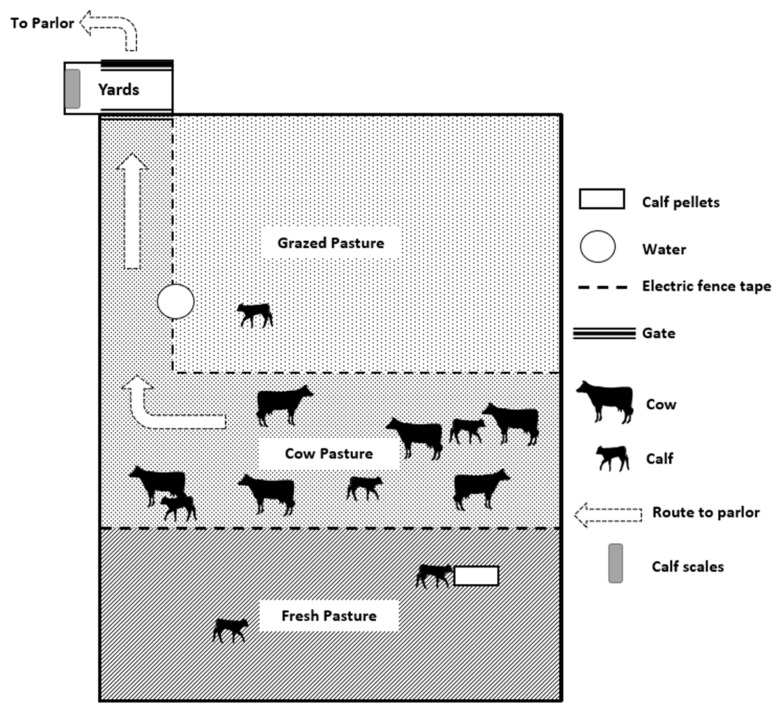
Farmlet design with route to remove cows from the paddock for milking is marked with arrows.

Cows and calves were moved from the calving paddock within 12 h of calving to a pasture-based system (farmlet), which was less than 500 m away. The farmlet was 330×40 m which opened into a set of yards (11×4 m) to ensure the safe separation of cow-calf pairs if required (Figure 1). Cow-calf pairs were marked with tail paint with a number (1 to 6) on their sides for identification. Cow and calf identification numbers were reapplied with tail paint as needed. Cow-calf pairs remained in the farmlet until full separation was initiated at 106±8.6 d and completed at 109±8.6 d.

Colostrum quality was determined at the first milking using an optical brix refractometer (% on Brix scale; E-line ATC Optical Refractometer, Kent, United Kingdom). Although all calves were able to suckle within x hours after birth, approximately 2 L of the mother’s colostrum (if the refractometer reading was greater or equal to 21% or ≥50 mg/mL of immunoglobulin G as per Quigley et al [11] was administered via esophageal feeding tube to the calf within 12 h of birth to ensure adequate colostrum consumption. For cow-calf pairs where the cow’s colostrum tested at first milking was below 21%, 2 L of frozen colostrum testing between 25% and 29% on the brix scale was thawed and administered via esophageal feeding tube to the calf. Calves also had their navel sprayed with iodine, were weighed using standard livestock scales (Thunderbird T30/2000; Thunderbird Ag Pty Ltd, Mudgee, Australia), and marked with a numerical ear tag within 24 h of birth. Calves were weighed once per week to determine average daily gain (ADG).

Cows were milked twice a day at 0400 h (morning milking) and 1230 h (afternoon milking) in a herringbone milking parlor (DeLaval, Botkyrka, Sweden) for the first 4 d, and then in a rotary, robotic milker (AMR mk1; DeLaval, Sweden) thereafter. At each milking, cows were temporarily separated from their calves for approximately 1 h. During the temporary separation process for milking, cows were herded away from their calves and into the yards to leave the paddock. For the first month of the study, two experimenters separated cows and calves, but this was manageable by one individual thereafter. The set of yards (Figure 1) was used as a sorting area to prevent calves from leaving the paddock. For the first 28 d, 1 kg/cow/d of concentrate was placed in feeding troughs in the yards as an incentive for the cows to leave the paddock for milking and to train the cows to the system. Any calf that entered the yard was drafted from the cows and returned to the pasture. Cows were then walked to the parlor to be milked. The duration of cow removal from the paddock was less than 10 min.

Cow and calf behavior were recorded via direct visual ob servations using a 0/1 binomial scan sampling method where behavior was either present (yes = 1) or absent (no = 0) with each observer monitoring 6 animals per observation period. The behaviors ‘close to calf’, nursing/suckling, vocalization, cow turn arounds, standing, and grazing were measured at specific timepoints as described by the ethogram in Table 1. Observations were conducted before and after the afternoon milking, between 11:30 and 14:00, for the first 7 d post-partum and then twice a week, at the same times, thereafter. Within each observation day, 6 time periods were recorded: i) Pre-milking: 15 min before separation for milking; ii) Temporary separation process: removing the cows from the paddock for milking; iii) Parlor herding: from the time the cows exited the yards until they entered the parlor; iv) Temporary separation: 15 min of calf observations after the cows exited the yards; v) Return: once the cows exited the parlor until they entered the paddock; and vi) Post milking: 15 min after all cows entered the paddock after milking. Each 15 min period consisted of 10×1 min 30 s intervals. Two observers monitored the 6 cow-calf pairs (3 pairs per observer) during pre-milking, temporary separation process and post milking. During that time, the observer would document the specific behaviors from 3 cow-calf pairs in numeric order (cow 1, calf 1, cow 2, etc) at the start of each interval. One observer monitored the 6 cows as they were moved to and from the milking parlor (parlor herding period and return period), while the other observer stayed in the paddock monitoring the 6 calves during the separation period.

Cow milk production, measured as daily milk yields, was recorded by inline individual, quarter milk meters (DelPro; Delpro Equipments Pty Ltd., Maharashtra, India) at each milking. Data were downloaded from DelPro (Delpro Equipments Pty Ltd., India) when the cows started to be milked on the rotary (4 d postpartum) until 3 d after weaning at 109±8.6 d. To estimate the amount of milk calves consumed around the time of separation, we subtracted the average milk yield of the 3 d directly before separation from that of the 3 d following separation.

### Feed management and monitoring

Farmlet pasture was comprised of 100% kikuyu (*Pennisetum clandestinum*) that was under sown to annual rye grass (*Lolium multiflorum*) on day 44 of the study period. Cows were maintained in the “Cow Pasture” (Figure 1) and pasture was allocated daily to cows using a strip grazing system. Two temporary fences prevented cows from entering the “Grazed Pasture” and the “Fresh Pasture” sections of the paddock. The temporary electric tape fence was moved daily to offer fresh pasture from the “Fresh Pasture” section of the paddock according to pasture growth rate with a second temporary tape preventing cows from overgrazing the previous day’s pasture allocation in the “Grazed Pasture” section of the paddock. Calves were able to access the entire paddock by going under the electric tape fence. When levels of pasture growth were lower than cow requirements, the diet was supplemented with lucerne hay or corn silage. Across the duration of the experiment, cows were offered on average 4.5 kg of pasture dry matter (DM). Supplemental lucerne hay or corn silage was offered at an average of 7 kg DM/d when required. During the first 47 d of the experiment, cows were allocated grain-based concentrate, from individual automated feeders (DeLaval, Sweden), based on their daily average milk production (kg/d). However, this milk yield did not account for the milk consumed by the calves. Therefore, from day 48 until the end of the study, cows were offered a fixed ration of 10 kg DM/cow/d in the individual feeders before returning to the paddock.

Calves had *ad-libitum* access to the fresh pasture and grain-based starter pellet (22% crude protein) provided in a feed trough that they could access by walking under the electric fence (Figure 1). Daily starter pellet refusal was weighed to calculate consumption. The average pellet consumption/calf/d across the entire study was 170 g/calf/d. The beginning of the study averaged 146 g/calf/d and increased to 214 g/calf/d.

### Carcass and gastrointestinal tract assessments

After 106±8.6 d, calves were fully separated from the cows and transported to a local abattoir (approximately 32 km from the farm) where they were processed within 24 h. Digestive tracts were collected from the abattoir and frozen for further analysis. Calf carcass weight was recorded, and meat and fat color were determined using the AUS-MEAT grading system whereby meat color increases in darkness from 1 (A–C) to 7 with consumers preferring meat between 1B and 3 [12]. Gastrointestinal tracts were collected from the abattoir, frozen and later dissected into the reticulorumen, omasum and abomasum. For every calf, each stomach compartment was laid on the left side and cut along the outer circumference and the content of each compartment discarded and rinsed with cold water [13]. Each stomach was then weighed (SK-20KWP; A&D PTY LTD, Thebanon, South Australia) and the proportion of the compartment weight determined relative to total stomach weight.

### Statistical analysis

The experimental unit, behavior, was categorized by observation period, weeks and stages of the experiment: early (1 to 28 d), mid (29 to 62 d) and late (63 to 84 d) for all behavior analysis. Behavioral data were analyzed using a generalized linear mixed model in GenStat 16 edition (VSN International, Hemel Hempstead, UK). Cow number/calf number was the random effect for each respective behavioral data analysis. Calf BW and milk yield were analyzed by experimental week using a restricted maximum likelihood linear regression in GenStat 16th edition (VSN International, UK). Significance was determined as p≤0.05.

## RESULTS

### Cow and calf behavior

The probability of each behavioral parameter (calf grazing, calf location, suckling, proximity to cow, calf and cow vocalizations, and cow attempts to return to her calf during milking) occurring within each observation period are shown in Table 2.

Calf grazing behavior was observed as early as week 1 (1.6%) and increased with week of the study with a peak of 26% of occurrence by week 13 (p<0.001). Calf grazing had higher probability during separation (13.9%±11.7%) and post-milking (11.6%±7.6%), as compared to pre-milking (8.6%±7.0%) (p<0.001).

During week 1 after birth, calves were two times more likely to be located within 2m of their mother as compared with other weeks. Calves were observed in the cow pasture (Figure 1) increasingly over time (p<0.001), with a mean proportion of time of 43.5%±0.08% across all weeks compared to the grazed pasture and fresh pasture. Calves were in the cow pasture more during pre-milking (49.4%±6.6%) and post-milking (51.6%±14.5%) as compared to during separation (29.4%±4.1%; p<0.001). Even though the time spent in the cow pasture increased over time, the time calves spent near their mother decreased.

Suckling occurred less than 10% of the observed time dur ing the pre- and post- milking periods and was more common post milking (7.0%±1.1%) than pre-milking (3.7%±3.8%; p<0.01).

Calf vocalization occurred in ≤10% of each observation period, however, there was a trend for higher rates of occurrence during the early (days 1 to 28) and late stages (days 63 to 84) of the study, as compared to the mid-stage (p<0.1).

Cow vocalizations were twice as frequent during the early stage (days 1 to 28) as compared to subsequent stages. The probability of a cow vocalizing during the temporary separation process (when separated from her calf for milking) was 39.2% during the early stage of the study, as compared to 26.5% during the later stage (p<0.05). Vocalizations were greater during herding to the parlor than all other observation periods, with a probability of 50.3% in the early stage and decreasing by nearly half by the later stage of the study (p<0.05).

Cows’ attempts to return to their calves occurred 5 times more frequently in the early stage of the study when compared to the mid and late stages. When cows were moved to the milking parlor the probability of a cow attempting to return to their calf declined from 30% during early stages to about 2% for mid and late stages of the study.

### Cow and calf performance

Milk yield averaged 11.2±5.6 kg/cow/d (recorded from 4 to 106±8.6 days in milk) while the calves had access to their mother. Individual mean weekly milk yields data are provided in Table 3. The average milk yield across 3 d after removal of the calf increased to 31.3±8.6 kg/d (p = 0.015), a difference of approximately 19±3.4 kg/d.

**Figure 2:**
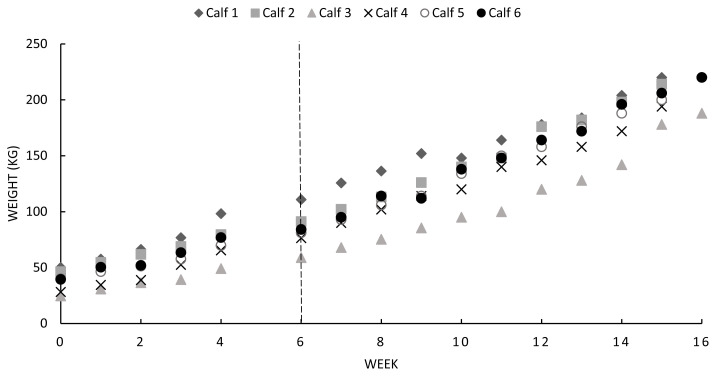
Individual calf weights (kg) from birth to separation. Calves doubled their birthweight at week 6, displayed by the dotted line (---).

Weekly calf BW are displayed in Figure 2. The mean calf birth weight was 42±4.3 kg, with calves doubling their birth weight by 6 wks of age. Average calf BW at weaning around 100 d of age was 206±13.9 kg, resulting in an average daily BW gain of 1.4±0.2 (ranging from 1.3 to 1.5) kg/d.

Mean calf carcass weight was 121.6±4.4 kg ranging from 110.8 kg to 137.0 kg. The mean carcass yield was 59.0%±2.7% of BW, ranging from 55.4% to 62.5%. Meat color and fat color averaged 1C and 0.7±0.2, respectively. The mean Meat Standards Australia marbling number was 250±22.4. Mean eye muscle area was 61.8±0.8 cm^2^.

Reticulorumen and abomasum weights averaged 2.06 (±0.14) kg and 0.9 (±0.1) kg, respectively. Reticulorumen weights varied from 1.7 kg to 2.7 kg. The reticulorumen and abomasum weight percentage of the total stomach weight was 54.9%±0.14% and 24%±2.2%, respectively.

## DISCUSSION

Calves spent almost half of their time in close proximity to their mother in the first week of life, but this declined rapidly as calves aged. Due to the limited work conducted over the long-term on dairy cow-calf systems (particularly those reared on pasture), extrapolation from cow and calf behavior in beef systems is used here for comparison. Greater distances between beef cows and their calves have been reported as calves aged [14]. In the study by Vitale et al [14], cows and calves were observed for 70 d postpartum, with calves spending the majority of time more than 15 m from their mother. In our study, calf independence increased with calf age, as indicated by the greater distance between calves and their mother and the decrease in suckling bouts. Suckling is a bonding as well as a nutritive behavior [15]. The calves in the current study decreased suckling frequency by 5% from wks 1 to 12, corresponding with increased consumption of other feeds. Similar reductions in the duration and number of bouts of suckling have been observed for pasture-reared beef calves from 1 to 6 mo of age [15], and for intensively reared dairy calves maintained on the cow for 8 weeks [16].

Grazing became more frequent as calves aged, increasing by 17% from wk 1 to 12. Work undertaken about 6 decades ago reported that male dairy calves reared on pasture without access to the cow began grazing at about three weeks of age [17]. The lack of more recent data suggests the need for future work evaluating cow and calf grazing behavior. Our findings are consistent with previous work reporting an increase in grazing when calves [18] were introduced to pasture with mature cows, suggesting that less experienced cattle may learn from older animals. Thus, rearing calves together with the mother may provide some learning and behavioral advantages to develop grazing behaviors at a younger age.

The cow-calf system in this experiment involved twice daily separation of cows from calves for milking, due to the impracticality of managing calves in the milking parlor. Most research describing the behavior of cows and calves at separation is limited to acute behaviors indicative of distress (mainly vocalizations and seeking behaviors) and investigating long-term behavioral effects in calves (e.g. abnormal and social behaviors) [10]. Cows and calves vocalize in response to separation distress [19]. The low frequency of this behavior in this experiment (average percentage of time vocalizing: 3% across the study) suggests that calves (and cows) quickly habituated to the routine. Similarly, the 5-fold reduction in attempts by the cows to return to their calves in the early stage of the experiment when compared to the mid and late stages, and the low probability of this behavior in the mid and late stages (2%) also suggests that cows habituated to temporary separation during milking. More work is needed to assess the intensity and duration of distress responses to temporary separation in cows and calves.

Cow vocalizations and attempts to return to their calf on the way to milking during the temporary separation process may provide some insight into the acute stress associated with temporary separation [19,20]. These responses declined rapidly as cows habituated to the system, however multiparous cows were used in the study, and it is unknown how first lactation heifers may respond. To our knowledge, this is the first experiment to describe vocalizations during rearing of calves and cows kept on pasture. The results are in line with the low number of vocalizations recorded during the first few hours after full separation in previous early separation studies in indoor housed systems [21,22]. The period of separation for milking (approximately 2 h) is similar to the first few hours of full separation despite our cows being habituated to the routine of temporary separation. Thus, the initial cow response to this short period of separation within the first few weeks of the experiment is comparable to the response within the first few hours of full calf separation. However, the longevity of the study allowed for habituation to temporary separation resulting in the decrease in stress response.

Attempts by the cows to return to the paddock while walk ing to the milking parlor reduced by 25% between week 1 and week 10 of the experiment. Similarly, although anecdotal, Grøndahl et al [23] stated that they observed little to no distress in response to temporary separation for milking when cows were kept with their calves in an intensive system for 6 to 8 wks; however, more work using larger sample sizes is needed to substantiate this claim. Incrementally enrolling cows into a cow-calf contact system might aid in habituating new cows to the system, as new members learn the routine through the habituated cows. The use of a grain reward when exiting the paddock may also assist the training process and can be removed once the cows have habituated to the system. Overall, our findings indicate that short term separation to allow for milking can be achieved, in the context of this experiment, and provides evidence that cows are able to habituate to the routine of this system.

Our results highlight the potential to achieve high levels of calf growth when they are offered unrestricted access to their mother, in addition to concentrate and pasture or hay in a pasture-based system. The elevated BW gains of these calves was likely due in large part to a high quantity of milk consumed, though actual milk consumption was unable to be measured. Based on the increase in cow milk yields after separation, we estimated calf milk intake at approximately 19 L/d in the final week of the study (about 15 wk of age) and corresponds to approximately 10% of their BW at this time which is the recommended milk consumption for dairy calves [6,7,24]. We were unable include a comparison group of non-nursing calves of a similar age and sex due to logistical constraints on farm, however these consumption estimations are greater (although comparable) than the 12 L/d at 9 wk reported by de Passillé et al [2], and the 15 L/d at 13 wk reported by Roth et al [25] where calves had *ad libitum* access to suckle from their mother. Our experiment consisted of only male calves which have been reported to drink 16 L/d at 6 wk of age [26] providing reasonable explanation for the 19 L/d at 15 wk. These results suggest that milk consumption of Holstein calves reared with their mother continues to increase about 1 L/wk from 9 to 15 wk after birth. However, calves allocated *ad libitum* milk replacer allowance was less than our calves with reported consumption between 8 L/d and 12 L/d [7,27]. According to Asheim et al [9], comparisons to *ad libitum* milk allowances using automatic milk feeders is difficult as calves may drink more while suckling from cows. However, increased milk consumption linked to higher milk yields in first lactation [9] suggest a pay off when these replacement heifers begin lactating.

Calf BW gains averaged 1.4 kg/d, more than double the industry standard of 0.6 kg/d for conventionally reared Australian heifer calves [24] and calves fed lower volumes of milk either individually or in groups (4 L/d of milk from wk 1 to 7; then 2 L/d, 0.68 kg/d [28]; 4L/d, 0.81 kg/d [29]). The gains we reported are double those of Holstein-Friesian calves reared conventionally which is expected given the restricted milk intake of conventionally reared calves compared to *ad-libitum* access to milk in the current study. Daily BW gains in our study were also higher than those reported for calves on greater than conventional milk allocations (6 L/d, 0.58 kg/d; 8 L/d, 0.57 kg/d; 10 L/d, 0.65 kg/d; and 12 L/d, 0.88 kg/d; [7]; 4 L/d of milk from wk 1 to 7, then 2 L/d, 0.68 kg/d; [28]; 8L/d, 0.86 kg/d; [29]) and calves fed *ad libitum* milk from artificial teats (0.82 kg/d, 6; 0.81 kg/d, [30]). The calf weight gains in the current study were more comparable to those of beef calves reared with cows on pasture, with ADG ranging from 1.3 to 1.5 kg/d [31]. The use of only male calves in this study may have resulted in an exaggerated impact on growth as male calves have been reported to gain more weight than heifer calves [32]. These results suggest that providing calves with the opportunity to obtain milk directly from the cow is beneficial. Although calves had *ad libitum* access to grain-based concentrate, the amount consumed was negligible (<1 kg/calf/d) suggesting weight gain was impacted more from other sources of feed. The calves may have benefited from access to the fresh pasture, especially the high protein of the upper stratum of the pasture sward [33], however further work is required to disentangle these effects.

Pasture consumption has previously been associated with yellow fat color in meat [34–36]. However, the mean fat color from calves reared on the cow in the current experiment was white which is more favorable from a meat grading perspective [34,36]. The white fat color was likely due to the young age of the calves at slaughter, where the degree of yellow fat color increases with age [34], and that a large portion of calves’ energy intake came from milk rather than grass. Interestingly, calf meat in the current study was scored as a bright cherry red color, contrasting previous studies reporting a pale red for veal arising from calves fed milk and grain-based concentrate (127 d-old calves; [35]), but aligned with the findings of Muir et al [37] that pasture reared cattle have meat of darker color. The 100-day-old calves in this study also produced similar carcass yields (59%) as 127-day-old milk-fed calves (56%; [35]). Overall, this work demonstrates the potential to achieve favorable carcasses with white fat and red meat from a pasture-based cow-calf system, though replication with larger sample sizes would be necessary to understand this effect on a commercial scale.

In addition to measuring carcass characteristics, the stom achs of each calf were dissected and weighed. The calves had a smaller proportion of reticulorumen, and a greater proportion of abomasum, as compared to 12 to 16-wk-old conventionally reared calves, with the reticulorumen and abomasum averaging 67% and 15% respectively of the stomach weight [38]. High milk intakes can reduce grain intake and slow rumen development [39]. However, despite consuming large volumes of milk the calves in the current experiment had heavier reticulorumens (2.1 kg) than those reported for 8-wk-old (0.93 and 1.0 kg; [30]), 9-wk-old (conventional: 1.6 kg; [8]), 10 wk old (starter without hay: 1.59 kg; starter with hay: 1.89 kg; [40]), and 12-wk old calves (1.9 kg; [41]). Reticulorumen weights in the current study were similar to values reported previously for 9-wk-old calves weaned in a milk step-down program (STEP: 2.2 kg; [8]) and 14-wk-old calves reared conventionally (2.3 kg; [42]) while expectantly weighing less than 20-wk-old calves (2.5 kg; [43]). In addition, abomasum tissue from the calves weighed more (0.9 kg) than the industry average 9-wk-old (0.57 kg and 0.71 kg; [8]), 10-wk old calves (starter without hay: 0.48 kg; starter with hay: 0.47 kg: [40]), 12-wk-old calves (0.7 kg; [41]), 14-wk-old [0.5 kg; [42]) and 20-wk-old calves (0.5kg; [43]). It should be noted that all the calves in the reviewed studies were reared on restricted milk feeding programs, in contrast to *ad libitum* access to milk in the current study. Ellingsen et al [44] also found that the abomasum of calves offered high milk volumes (6 L per feeding) were more distended and larger than those fed restricted levels of milk (2 L per feeding). A distended abomasum is expected to be accompanied by slower rumen development, but this was not observed in the study perhaps associated with access to fresh pasture and the age at slaughter. Despite negligible starter pellet consumption, rumen development was on par with recommendations suggesting the need to re-evaluate the recommendations on grain calf starter in pasture-based dairy systems.

This study only evaluated 6 cow-calf pairs, which limits the conclusions of the study and extrapolation to a commercial level while providing direction for future research. The sample size provided the ability for a large data set and longitudinal data while also limited due to practicality and farm requirements. However, recent scrutiny against management of male dairy calves has encouraged farmers to grow out their male calves which gives dairy farms the opportunity to capitalize on meat market [45]. Future research using heifer calves can evaluate impact on longitudinal effects of this system through to lactation. Further research should include a treatment group of non-suckling cows and calves to allow for comparison of the impact of long-term calf suckling on production outcomes. Currently, there is an abundance of literature cow-calf separation in indoor housed systems, however there are clear gaps in knowledge as to the impacts of temporary separation and long-term behavioral responses of cows and calves when maintained together. The next probable comparison is beef cattle as they are maintained in similar environments (long-term cow-calf system on pasture) and natural cow-calf behavior has been consistent [46]. The use of historic research further identifies areas needing further research.

## CONCLUSION

Our experiment demonstrated some potential benefits of rearing calves on the cow in pasture based dairy systems. Calves and cows appeared to habituate to temporary separation for milking. In addition, social facilitation of social and grazing behaviors from the older, experienced cows to calves may be a potential advantage of this rearing system. Male calves reared on pasture together with their mothers showed high weight gains and good carcass characteristics. Cows reared with their calves produced lower milk yields during the rearing period, which increased rapidly after separation. Rearing calves together with cows in a pasture-based systems shows promise with these results providing an important basis for future work on pasture-based cow-calf rearing systems at a larger scale.

## IMPLICATIONS

We investigated a novel calf pasture rearing system focusing on the long-term effects on cow on production, behavior and growth. Cows and calves habituated to the system determined by the increasing time calves spend grazing and away from the cow, decreasing suckling events, cow vocalizations, and cows attempting to reunite with their calf. Calves gained weight twice the rate of industry standard before full separation and cow milk production rebounded rapidly after calf separation. The calves produced favorable carcass characteristics and achieved target rumen development. Through the results of this study, potential advantages to rearing cows and calves together on pasture are introduced providing a foundation for future research.

## Figures and Tables

**Table 1:** Ethogram with the description of cow and calf behaviors measured during live observations categorized by observation period and modified from Flower et al [22] and Weary and Chua [21]

Behavior	Definition	Observation periods
Close to calf	Cow positioned within one cow body length (2 m) of her own calf	Pre-milking, Post milking
Nursing	Cow’s calf has his nose or mouth in contact with mother’s udder followed by sucking on a teat with mouth	Pre-milking, Post milking
Turn arounds	Cow turns her head, neck, and the front of her chest oriented toward calf location	Temporary separation process, Parlor herding
Vocalization	Audible sound coming from animal’s mouth	Pre-milking, Temporary separation process, Parlor herding, Temporary separation, Post milking
Standing	Animal’s torso is not in contact with ground, all weight supported by hooves (includes standing still and moving (e.g. walking, running) while in a non-recumbent position	Pre-milking, Temporary separation, Post milking
Grazing	While standing, the animal has its head angled down (below withers) and moving muzzle (nose and mouth) along close to grass (within 10 cm) and taking grass into the mouth, followed by chewing	Pre-milking, Temporary separation, Post milking
Suckling	Calf having nose or mouth in contact with an unrelated cow’s udder followed by sucking on a teat with mouth	Pre-milking, Post milking

**Table 2:** Probability of cows and calves displaying the listed behaviors, when maintained together for 100 days, categorized by time and observation period

Behavior	Observation	Time period^1)^		

		Early	Mid	Late
Calf behaviors				
Vocalizations***	Pre-milking	0.6^a^	0.2^b^	2.1^c^
	Temporary separation process	9.7^a^	3.9^b^	10.0^a^
	Temporary separation	4.3^a^	1.9^b^	6.1^a^
	Post milking	4.1^a^	2.1^b^	2.5^b^
Grazing*	Pre-milking	2.5^a^	7.2^b^	16.2^c^
	Temporary separation	2.4^a^	13.4^b^	25.8^c^
	Post milking	3.0^a^	17.6^b^	14.1^c^
Cow pasture*	Pre-milking	48.2^a^	43.5^b^	56.5^c^
	Temporary separation	32.7^a^	30.7^a^	24.9^b^
	Post milking	36.7^a^	52.4^b^	65.6^c^
Close to Cow*	Pre-milking	38.1^a^	26.1^b^	22.3^c^
	Post milking	24.0^a^	17.0^b^	27.9^c^
Suckling*	Pre-milking	8.0^a^	1.8^b^	1.2^c^
	Post milking	7.5^a^	5.7^b^	7.8^a^
Cow behavior				
Vocalizations**	Pre-milking	11.8^a^	7.1^b^	4.8^c^
	Temporary separation process	39.3^a^	35.1^a^	26.5^b^
	Parlor herding	50.3^a^	35.1^b^	28.8^b^
	Return	32.4^a^	12.9^b^	15.3^b^
	Post milking	17.0^a^	4.7^b^	2.6^c^
Turn Arounds***	Temporary separation process	17.6^a^	11.1^a^	4.4^b^
	Milk herding	29.7^a^	2.2^b^	2.2^b^

1) Early, Weeks 1 to 28 d; Mid, weeks 29 to 62 d; Late, weks 63 to 84 d.

a–c Means within a row with different subscripts differ (* p<0.001, ** p<0.05, *** p<0.1).

**Table 3:** Weekly individual daily milk yields of cows maintained with their calves for 14 weeks and daily milk yields around time of separation (L/cow/d)

Cow number	Weeks														Days to separation^1)^					
	
	1	2	3	4	5	6	7	8	9	10	11	12	13	14	−3	−2	−1	0	1	2
Cow 1	7.2	12.4	15.0	12.4	10.2	4.4	5.9	8.1	19.2	21.6	15.2	12.0	10.7	10.8	2.9	15.2	24.3	33.3	28.0	42.0
Cow 2	8.8	14.1	14.2	14.5	13.6	6.7	6.9	10.2	11.0	13.6	10.5	12.8	10.7	11.9	10.7	9.5	13.6	28.5	21.8	31.1
Cow 3	3.7	5.9	5.8	6.4	4.2	5.6	6.6	9.8	8.9	8.8	7.9	7.4	6.2	8.7	6.3	5.3	5.8	21.0	19.6	20.5
Cow 4	10.2	15.0	15.0	11.8	10.1	12.8	11.4	17.8	15.1	15.5	16.8	15.5	19.4	18.4	22.7	16.8	32.5	40.4	40.1	51.5
Cow 5	7.6	13.5	11.0	9.1	5.5	6.8	9.8	6.7	9.5	9.2	8.2	8.2	6.9	7.8	8.7	9.4	8.9	30.6	28.8	39.5
Cow 6	6.4	12.7	13.3	13.7	15.4	18.2	17.0	14.7	14.9	15.7	14.6	14.1	14.9	14.6	10.4	7.2	13.1	30.5	25.8	30.6
Average^2)^	8.0	13.5	13.7	12.3	11.0	9.8	10.2	11.5	14.0	15.1	13.1	12.5	12.5	12.7	11.1	11.6	18.5	32.7	28.9	38.9

1) Day of separation = 0

2) Cow 3 was excluded from the average milk yields due to low milk yields resulting from premature calving.
